# SMASH – semi-automatic muscle analysis using segmentation of histology: a MATLAB application

**DOI:** 10.1186/2044-5040-4-21

**Published:** 2014-11-27

**Authors:** Lucas R Smith, Elisabeth R Barton

**Affiliations:** Department of Anatomy and Cell Biology, School of Dental Medicine, University of Pennsylvania, Philadelphia, PA USA; Pennsylvania Muscle Institute, University of Pennsylvania, Philadelphia, PA USA

**Keywords:** Histological muscle analysis, Standardized quantitative analysis, Image segmentation, *mdx* mouse

## Abstract

**Background:**

Histological assessment of skeletal muscle tissue is commonly applied to many areas of skeletal muscle physiological research. Histological parameters including fiber distribution, fiber type, centrally nucleated fibers, and capillary density are all frequently quantified measures of skeletal muscle. These parameters reflect functional properties of muscle and undergo adaptation in many muscle diseases and injuries. While standard operating procedures have been developed to guide analysis of many of these parameters, the software to freely, efficiently, and consistently analyze them is not readily available. In order to provide this service to the muscle research community we developed an open source MATLAB script to analyze immunofluorescent muscle sections incorporating user controls for muscle histological analysis.

**Results:**

The software consists of multiple functions designed to provide tools for the analysis selected. Initial segmentation and fiber filter functions segment the image and remove non-fiber elements based on user-defined parameters to create a fiber mask. Establishing parameters set by the user, the software outputs data on fiber size and type, centrally nucleated fibers, and other structures. These functions were evaluated on stained soleus muscle sections from 1-year-old wild-type and *mdx* mice, a model of Duchenne muscular dystrophy. In accordance with previously published data, fiber size was not different between groups, but *mdx* muscles had much higher fiber size variability. The *mdx* muscle had a significantly greater proportion of type I fibers, but type I fibers did not change in size relative to type II fibers. Centrally nucleated fibers were highly prevalent in *mdx* muscle and were significantly larger than peripherally nucleated fibers.

**Conclusions:**

The MATLAB code described and provided along with this manuscript is designed for image processing of skeletal muscle immunofluorescent histological sections. The program allows for semi-automated fiber detection along with user correction. The output of the code provides data in accordance with established standards of practice. The results of the program have been validated using a small set of wild-type and *mdx* muscle sections. This program is the first freely available and open source image processing program designed to automate analysis of skeletal muscle histological sections.

## Background

Skeletal muscle has a robust ability to adapt to the pattern of use and to regenerate following injury. These are often quantified using histological techniques. However, the methods for this quantification remain disparate among investigators and often require painstaking manual procedures [1, 2]. The goal of this work is to provide a widely available image processing software package specifically designed for muscle histological analysis.

Altering muscle fiber size is one of the primary methods in which muscle responds to external stimuli. Muscle mass may be increased in response to resistance training [3] or with potential pharmacological agents like myostatin inhibitors [4], while muscle atrophy occurs in response to disuse [5] and injuries such as denervation [6]. These conditions primarily reflect hypertrophy or atrophy of individual fibers rather than hyper- or hypoplasia [7]. Muscle fiber size is routinely evaluated using fixed or frozen tissue sections. Fiber outlines are visualized using a variety of techniques, including hematoxylin and eosin staining, laminin immunostaining, dystrophin immunostaining, and wheat germ aggluttinin staining [8]. While these techniques enable visualization of fiber boundaries, determining fiber cross-sectional area (CSA) is often still performed by manual tracing of individual fibers. There are software programs available to help automate fiber detection, however they are often expensive and are not specifically designed for muscle histology [9].

Muscle fiber type distributions are often investigated in muscle histology as they are known to be altered in response to exercise, inactivity, and aging [10]. Fiber type is primarily determined by the myosin heavy chain isoform, which has differential contractile and ATPase activity. Fiber type is often determined by ATPase staining [11, 12] or with immunostaining for specific myosin heavy chain (MyHC) isoforms individually [13, 14]. However, methods to determine fiber type can be subjective and tedious when fibers are manually classified. Following fiber segmentation, computing the size distribution of single fiber types is easily automated.

Muscle fibers also undergo changes in morphology as they develop. In particular, centrally nucleated fibers are often used as a marker for muscle regeneration. While fully mature fibers have peripheral nuclei, newly regenerated fibers have central nuclei [15]. In many muscular dystrophies, which are characterized by continual cycles of degeneration and regeneration, the number of centrally nucleated fibers (CNFs) is substantial while CNFs are hardly present in healthy muscle. Although nuclei are easily stained with DAPI, determination of CNFs is often performed manually. Combined use of automated CNF and fiber size determination allows the size of regenerating fibers to be calculated, providing a measure of how efficiently regeneration is occurring after acute injury [16].

Skeletal muscle is a highly metabolically active tissue requiring large blood supply. As with fiber type shifts, capillary density of skeletal muscle may be affected by altered metabolic demand or in disease [17]. Endothelial cells and capillaries are frequently stained in skeletal muscle with Von Willibrand Factor or PECAM [18, 19]. Automated determination of capillary density in relation to fiber size and number provides a useful parameter of skeletal muscle histology.

All of the methods discussed above are commonly performed using immunofluorescence, which provides high contrast in stained and unstained structures. We have developed MATLAB (MATLAB and Image Processing Toolbox 2014a, MathWorks) scripts bundled into a MATLAB App (see Availability and Requirements) that automate, or partially automate determination of fiber size, fiber type, centrally nucleated fibers, and capillary density. These programs are created to comply with standard operating procedures developed by TREAT-NMD when available using sophisticated boundary detection algorithms [2]. The software also includes built-in image editing to manually inspect and manipulate fiber boundaries. Fully automated fiber size determination as well as fiber types and CNFs may be possible with adequate image acquisition [9, 20]. However, these newly designed fully automated programs are not yet available [9] and/or have a significant cost [20]. Additionally, allowing the user to have manual control over some aspect of image processing allows users to maintain the fidelity established by manual techniques. The open nature of this software also allows custom usage and further advancement of the methods. For users that do not have access to a MATLAB license or the image processing toolbox we have compiled an .exe file that runs using the freely available MATLAB Runtime Compiler (MCR) version 8.3 (http://www.mathworks.com/products/compiler/mcr/). Automating a large portion of muscle histology makes it feasible to analyze full muscle cross-sections, eliminating variability introduced by selecting only a portion of the cross section for analysis. This software is validated with muscles from *mdx* mice, which have many alterations of muscle fiber morphology compared to wild-type mice [21]. The purpose of this study is to develop freely available automatic and standardized image segmentation platform and validate the program using standard muscle histological analysis.

## Implementation

### Mice

All animal experiments were approved by the University of Pennsylvania Institutional Animal Care and Use Committee. C57Bl/6 mice were used as wild-type controls and *mdx* mice were used as a dystrophic model. Both animal groups were analyzed at 1 year of age.

### Immunohistochemistry

Soleus muscles from both groups (n = 4 per group) were dissected, embedded in OCT, and frozen in liquid nitrogen cooled isopentane. Frozen 10 um sections were cut from muscles and mounted on slides. Sections were washed in PBS and immunostained using antibodies to laminin (Thermo Scientific), and either myosin heavy chain I (Developmental Studies Hybridoma Bank) or platelet endothelial cell adhesion molecule (PECAM; eBiosciences) overnight at 4°C. After PBS wash, fluorescent secondary antibodies (Sigma) were applied for 1 h at room temperature. Nuclei were labeled with DAPI incorporated into the mounting media (Vectashield). Images were acquired using a Leica DM RBE microscope and DFC350FX camera and OpenLab software. Individual fields were stitched together to create a composite full view of the muscle cross-section using Photoshop (Adobe).

### Image selection

**Figure 1:**
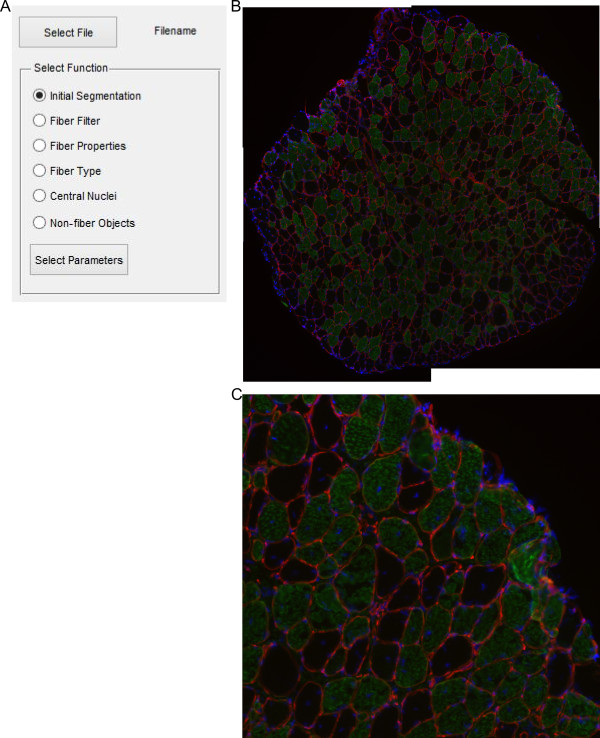
**Image selection. (A)** Dialog box allowing selection of a single function to perform on the selected muscle section. **(B)** Example muscle section of 1-year-old soleus muscle from *mdx* mouse. Red is laminin stain, green is slow muscle myosin heavy chain, and blue is DAPI. **(C)** Enlarged portion of **(B)**.

The software has built in several steps of image processing tools within the same script. Initially the user must select an image file (.bmp, .jpg, .png, and .tif.) to be processed. Following selection of the appropriate file the user is provided a list of the built in functions (Figure 1A). A representative image of a soleus muscle from a 1-year-old *mdx* mouse is used which has been immunostained with laminin (red) and slow myosin heavy chain (green) as well as DAPI (blue) (Figure 1B and C). Dystrophic muscle can be more difficult to process automatically due greater interstitial spaces and the examples highlight some manual adjustments that may be required. The software includes an Excel (Microsoft) file containing default parameter values (Table 1), which may be altered to the needs of the user. The details of each parameter are discussed in the relevant section below.

**Table 1:** **Default parameters from Excel file**

Default parameters	Default values
Pixel size (μm/pixel)	0.645
Fiber outline channel (red = 1, green = 2, blue = 3)	1
Nuclei channel (red = 1, green = 2, blue = 3)	3
Fiber type channel (red = 1, green = 2, blue = 3)	2
Object channel (red = 1, green = 2, blue = 3)	2
Segmentation smoothing factor	5
Nuclear smoothing factor	5
Object smoothing factor	10
Minimum fiber area (μm^2^)	100
Maximum fiber area (μm^2^)	5,000
Maximum eccentricity	0.95
Minimum convexity	0.8
Nuclear distance from boarder (μm)	10
Minimum nuclear size (μm^2^)	5
Fiber properties output folder	C:\SMASH\Output
Fiber type output folder	C:\SMASH\Output
Central nuclei output folder	C:\ SMASH\Output
Objects output folder	C:\ SMASH\Output

### Initial segmentation

**Figure 2:**
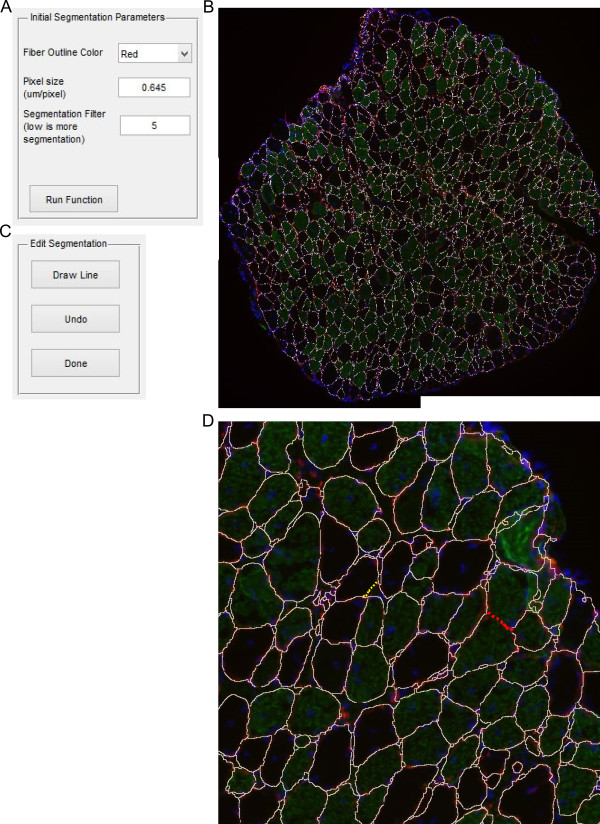
**Initial segmentation. (A)** Input box with options for fiber segmentation. **(B)** The example image with white lines drawn along fiber boarders based on segmentation parameters. **(C)** Dialog box that appears after separating a fiber. **(D)** Zoomed in of B. Red dotted lines shows where two fibers are separated by the user. Yellow dotted lines shows where a fiber is not separated from intestinal space.

**Figure 3:**
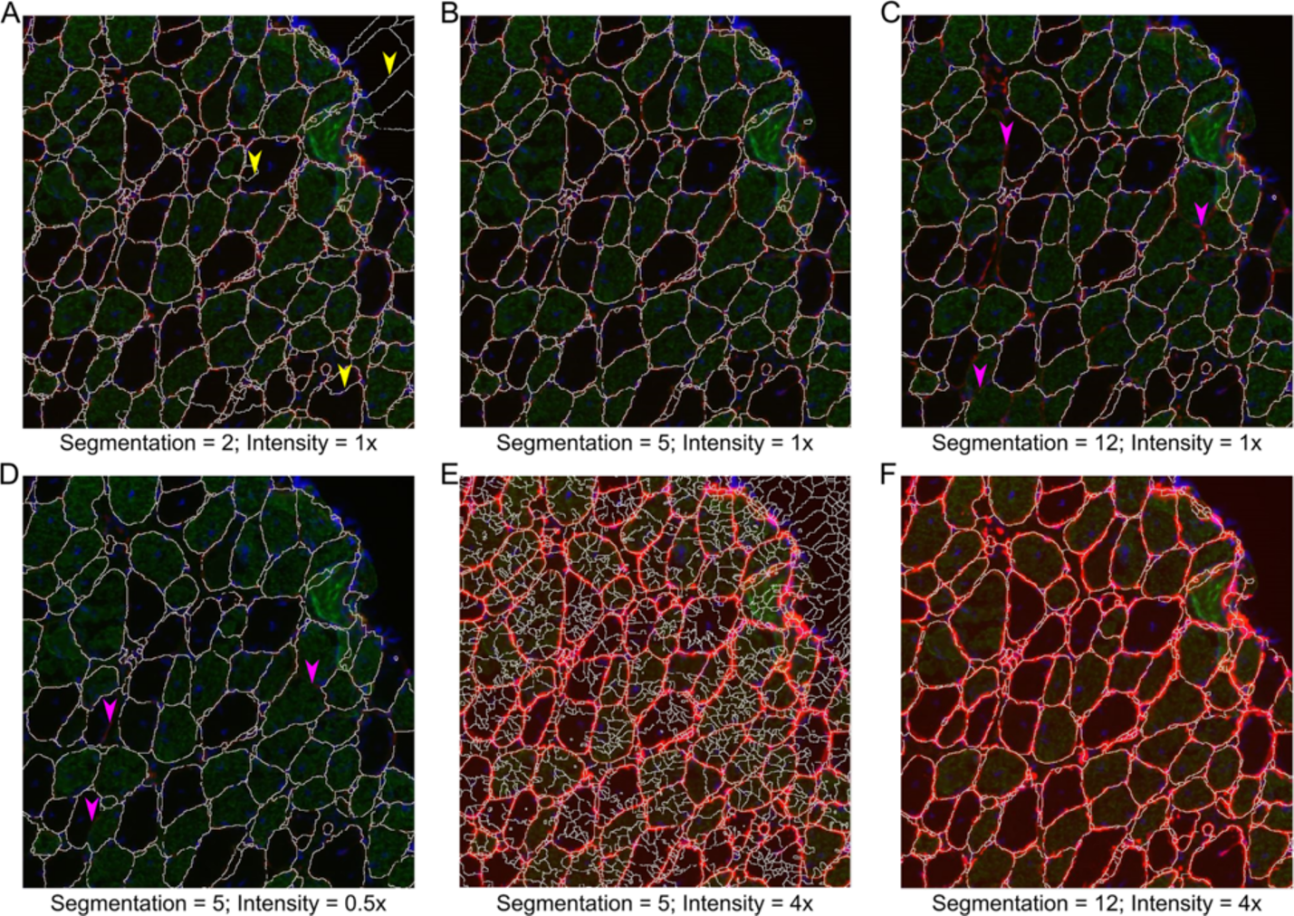
**Segmentation examples.** Example muscle section of 1-year-old soleus muscle from *mdx* mouse. Red is laminin stain, green is slow muscle myosin heavy chain, and blue is DAPI. **(A)** Segmentation filter value of 2 produces over segmentation of original image. **(B)** Segmentation filter value of 5 produces appropriate segmentation. **(C)** Segmentation filter value of 12 produces under segmentation of original image. **(D)** Reducing the brightness of laminin staining by 50% (representing poor staining) produces under segmentation. **(E)** Enhancing the brightness of laminin by 400% (representing over exposure) produces over segmentation. **(F)** Overexposure can be compensated for by increasing the segmentation filter value to 12. Yellow arrow heads show areas of over segmentation. Pink arrow heads show areas of under segmentation.

Prior to any other function being run, a mask file for the image must be created with the ‘Initial Segmentation’ function. The Initial Segmentation function uses the watershed transformation to determine the fiber edges, as it has become one of the most common and standard choices for image segmentation [22, 23]. However, the watershed transformation often leads to over-segmentation due to local minima created from noise within an image [24]. Prior to applying the watershed transformation, the image is smoothed with a function that suppresses local minima whose depth is below a given threshold. Upon selecting this function users are presented with options for segmentation (Figure 2A). The pixel size measured in μm/pixel is requested in all functions. The segmentation takes place based on a single RGB color channel, which the user provides designating the color of the fiber outlines. The user also provides an initial value for suppressing local minima based on 8-bit channel images (0 to 255). Previous experience shows that a segmentation value of 4 to 10 produces robust fiber identification, depending on the quality of staining (Figure 3). However, the segmentation value may need to be adjusted based on the image quality and exposure (Figure 3). For example, a suboptimal segmentation can generate false objects (Figure 3A, E) or merge objects (Figure 3C, E). Once initial parameters have been selected, the initial segmentation takes place with the segmented fiber edges overlaid on the original image (Figure 2B) [25]. After inspecting the image the user can decide to re-run the function with alternative parameters. Once proper segmentation has been achieved, the user has the option to separate any merged fibers that may have arisen from poor staining. The user implements a freehand drawing tool to separate any fibers that are merged with other fibers (red dotted line) or interstitial space (yellow dotted line) (Figure 2C). After the user has finished separating any fibers a mask file is saved. Initial segmentation will often produce many small segments in interstitial space; however, non-fiber areas will be filtered in the ‘Fiber Filter’ function. All fibers in the mask should be separated prior to running the fiber filter function to prevent inappropriate filtering of fibers.

### Fiber filter

**Figure 4:**
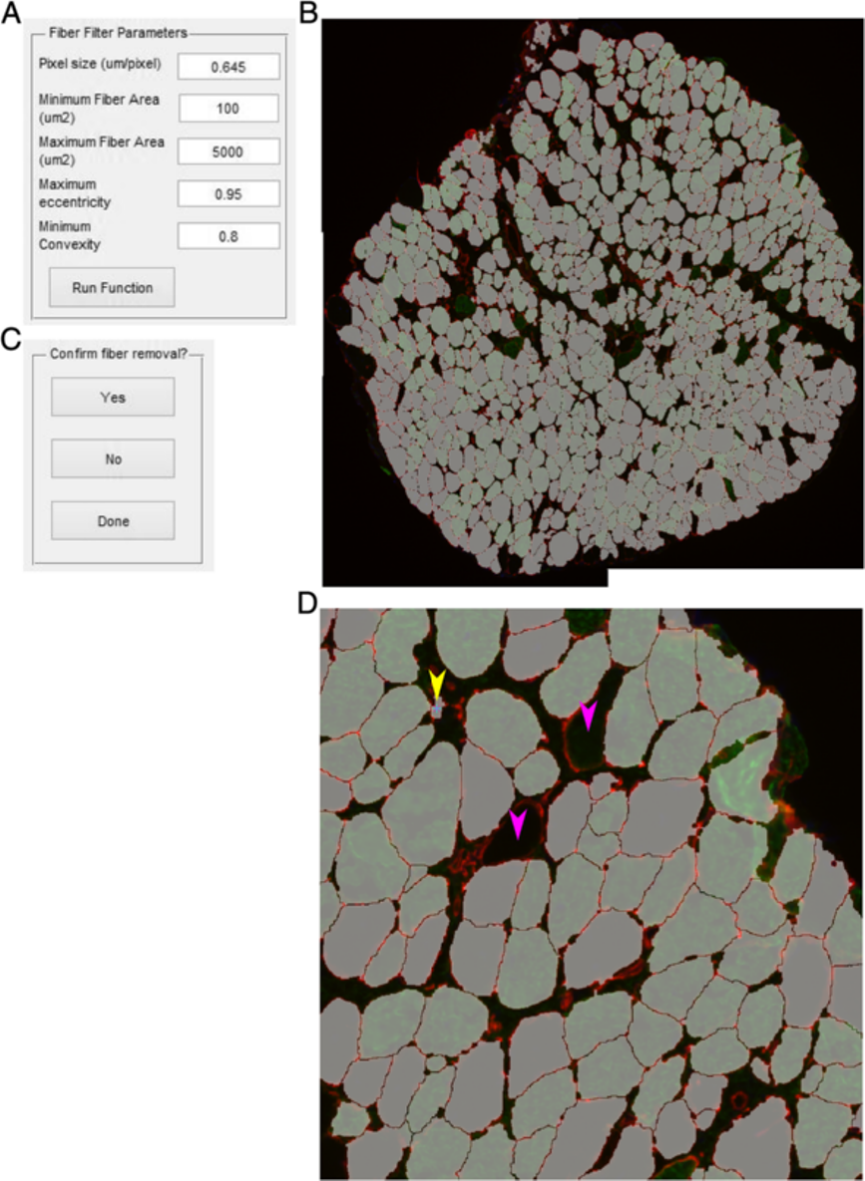
**Fiber filter. (A)** Input box with options for filtering fibers. **(B)** Image generated in which fibers passing the filter are randomly colored to more easily distinguish adjacent fibers. **(C)** Zoomed in of **(B)**. Yellow arrows point to possible user selections as non-fiber objects. Pink arrows show fibers that did not pass the filter. Lower left pink arrow did not pass due to improper segmentation as depicted in Figure 2D. **(D)** After selection of fibers the user is asked if they would like to indeed delete them or complete the function operation.

Following the initial segmentation the fiber filter function removes objects based on user-defined criteria (Figure 4A). Fibers that touch the edge of the image are removed so partial fibers are not included in further calculations. To provide the proper scale, the pixel size is entered by the user in μm/pixel. Any fibers below the minimum fiber area or above the maximum fiber area entered in μm^2^ are removed from the mask and from further analysis. More advanced filtering is also included with eccentricity and convexity. Interstitial space may fit the size requirements of a fiber; however, it will not have a grossly circular shape in cross section. The eccentricity takes an ellipse with the same second moments as the objects and is the ratio between the distance between the foci of the ellipse and the major axis. Thus, circles have an eccentricity of 0 and line segments have an eccentricity of 1. Further, interstitial space may have a stellate shape between fibers while maintaining high eccentricity. The stellate shape creates regions of concavity, while polygonal fibers have minimal concavity and high convexity. The convexity is determined by the ratio of the object area and the smallest convex polygon that can contain the entire region. Thus, a completely convex image will have a convexity of 1. After the filtering parameters have been selected, a new mask is created with only the objects passing the filter (Figure 4B) [25]. At this point the user has the option to inspect the mask and select any objects that may have passed the filter but are not fibers (Figure 4C). This may include areas of interstitial space, vessels, or other structures the user does not want to include (Figure 4D, yellow arrow). The zoomed example image shows fibers that were filtered because of high eccentricity and high convexity due to improper separation of fiber from interstitial space (Figure 4D, pink arrows). Following user removal of non-fiber objects the mask file is overwritten.

### Fiber properties

**Figure 5:**
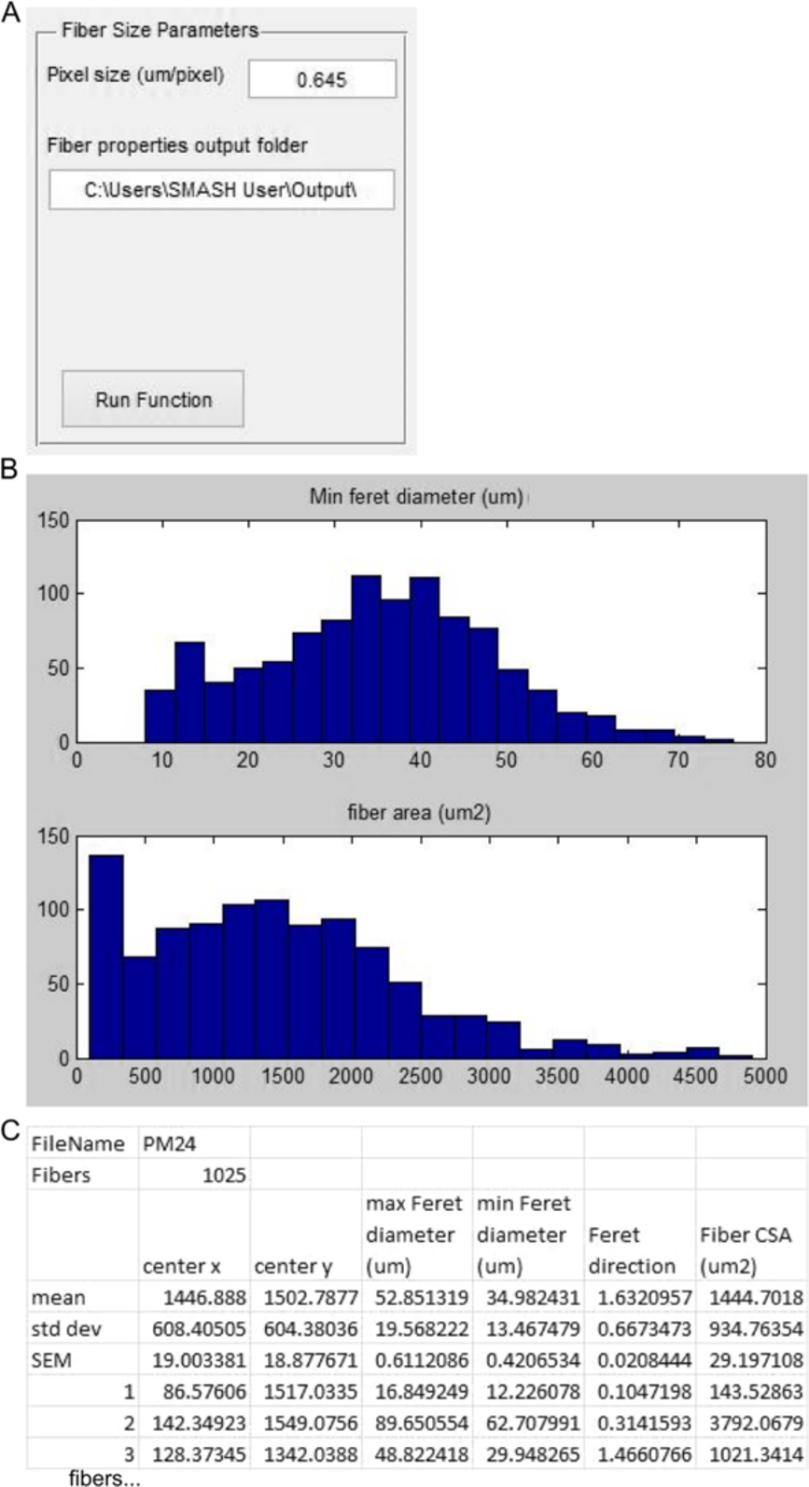
**Fiber properties. (A)** Input box with options for fiber properties. **(B)** Histograms showing minimum Feret diameter (top) and fiber CSA (bottom). **(C)** Truncated example of Excel output from running fiber properties function.

After the mask has been filtered to contain only objects that are fibers the properties of the fibers may be determined. The user is prompted for pixel size and the folder location that the output data should be exported to. The output file is in Excel (.xls) format with the same name as the image file appended by ‘_Props’ (Figure 5A). The Feret diameter properties are obtained from an incorporated publically available script on MATLAB Central [26]. The function displays histograms for fiber minimal Feret diameter and fiber CSA for inspection (Figure 5B). The Feret properties are defined by the smallest rectangle that bounds the fiber [27]. The minimum Feret diameter has been shown to be a more robust measure of fiber size. Feret diameter is much less sensitive to oblique muscle section compared to fiber CSA [2]. For each fiber the properties exported are: centroid in x and y in pixels, the maximum Feret diameter (μm), minimum Feret diameter (μm), Feret direction (radians), and fiber area (μm^2^). The output Excel file is then written with all fiber properties as well as the mean, standard deviation (SD), and standard error of the mean (SEM) for each property (Figure 5C).

### Fiber typing

**Figure 6:**
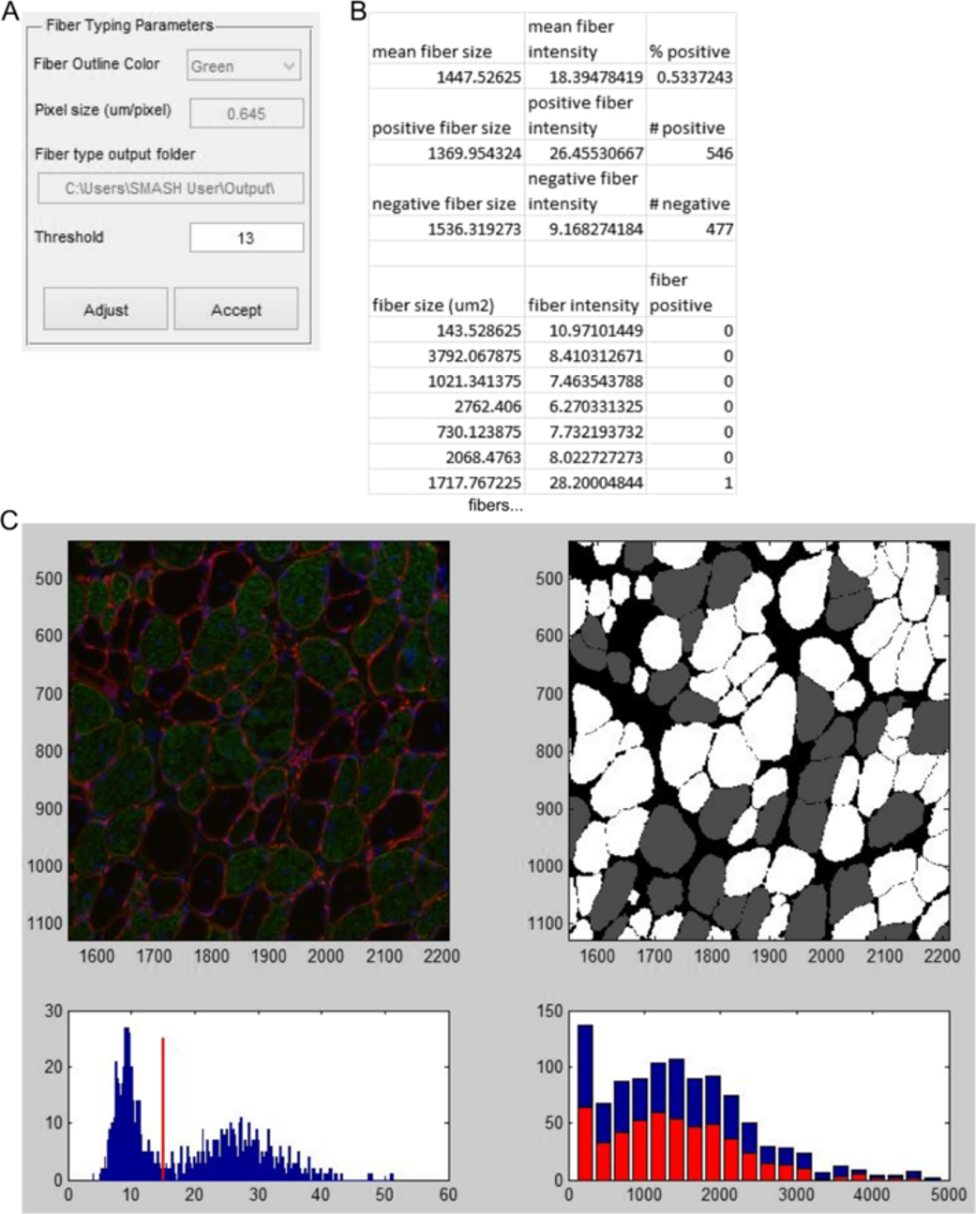
**Fiber type. (A)** Input box with options for fiber type function. **(B)** Truncated example of Excel output from running fiber type function. **(C)** Portion of immunofluorescent image. Red is laminin stain, green is slow muscle myosin heavy chain, and blue is DAPI (top left). Image showing negative fibers in gray and positive fibers in white (top right). Histogram of average fiber intensity for fiber type stain with threshold value (bottom left). Histogram of fiber CSA with positive fiber in red and all fibers in blue (bottom right).

The fiber typing function uses the fiber mask to inspect each fiber area for determination of fiber staining. The function examines the average intensity of staining within the individual fiber region determined by the mask to calculate an average intensity. A threshold value is used to determine if the fiber is positive or negative based on the average intensity. The user inputs the pixel size, the RGB channel which includes fiber type staining, and the output folder location (Figure 6A). The initial threshold value for average fiber type intensity is obtained using Otsu’s method, which calculates the threshold to minimize intra-class variance of the thresholded binary image [28]. The program then performs an initial calculation of fiber types. A figure is displayed with the original image and an image where positive fibers are white and negative fibers are grey (Figure 6D). The figure includes a histogram of staining intensities along with the threshold. The intensity histogram should have two peaks, with the lower and sharper peak being negative fibers and high intensity peak representing positively stained fibers. Intermediate values may represent mixed fibers. A histogram with the CSA of positive fibers (red, front) and all fibers (blue, back) is presented to examine any fiber type-dependent size changes. After inspecting the figure the user is able to adjust the threshold value and the figure is updated. Once the user is satisfied with the analysis they select accept and are prompted to create an Excel file with each fiber’s average intensity, fiber area, and if it is classified as positive (1) or negative (0) (Figure 6B). Summary data including average fiber area, average fiber intensity and percent of positive fibers are included for all, positive, and negative fibers.

### Centrally nucleated fibers

**Figure 7:**
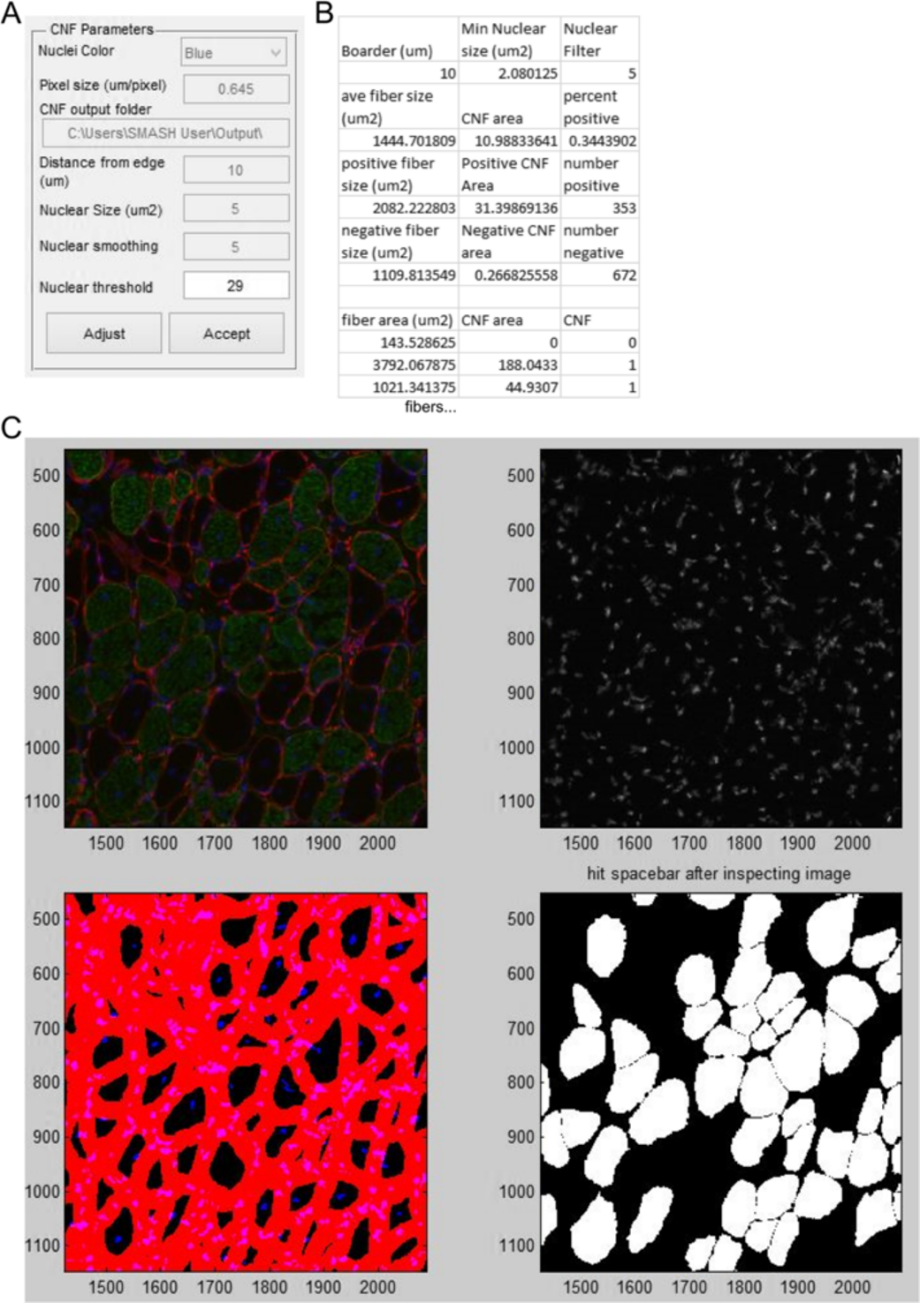
**Centrally nucleated fibers. (A)** Input box with options for CNF function. **(B)** Truncated example of Excel output from running CNF function. **(C)** Portion of immunofluorescent image. Red is laminin stain, green is slow muscle myosin heavy chain, and blue is DAPI (top left). Image showing filtered nuclei (top right). Image with boarder regions in red and nuclei above threshold in blue (bottom left). Image in which only CNFs are depicted in white (bottom right).

The CNF function again requires prior creation of a fiber mask to analyze the centers of fibers for nuclei. The user is prompted to input pixel size as well as the RGB channel that corresponds to nuclear staining (Figure 7A). To restrict the inspection to central nuclei the nuclear staining must be greater than a user-defined distance from the border of the fiber input in μm. The user is also allowed to specify the minimum area of nuclei to count so that random staining is minimized. To minimize the effect of streaking that can be present with poor DAPI staining the image can be smoothed using the same suppression of local minima used for fiber segmentation. An automatic threshold is applied after nuclei smoothing using Otsu’s method. Finally, the user also specifies the output folder for the Excel data to be written to. After input selection a figure is displayed that contains the original image and an image with the borders. The distance not inspected is in red and the nuclei are in blue, with peripheral nuclei appearing purple (Figure 7C, bottom left). The figure also contains images of only the smoothed nuclear image and an image with only CNFs. In some situations the automatic thresholding of nuclei may be unsuitable. After inspecting the image the user may adjust the threshold value as desired. Mousing over smoothed nuclear image may provide useful information for manual thresholding. Once a proper threshold is determined, the user is prompted if to determine if the results should be output to Excel. If the answer is yes, a file is created that contains the fiber area, the area of positive nuclei staining in the center region, and if the fiber is classified as a CNF (1) or not (0) (Figure 7B). Summary data include the average fiber area, area of central nuclei, and percent of CNFs for all fibers, CNFs, and non-CNFs. If parameter selection was not sufficient, the function can be rerun with new parameters.

### Capillary density or non-fiber objects

**Figure 8:**
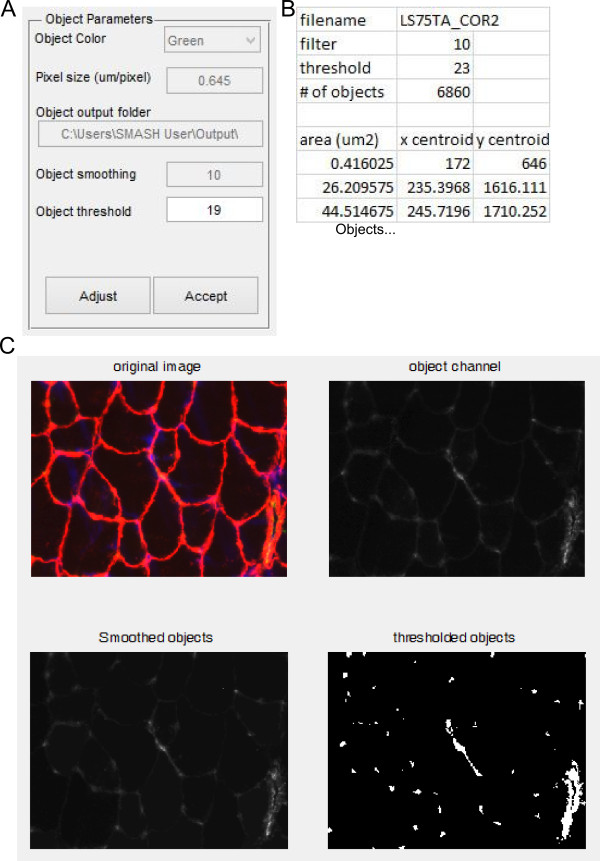
**Object counter. (A)** Input box with options for object counter function. **(B)** Truncated example of Excel output from running object counter function. **(C)** Portion of immunofluorescent image. Red is laminin stain, green is PECAM, and blue is DAPI (top left). Image showing only the objects of interest, here PECAM from the green channel (top right). Image showing objects of interest after smoothing filter is applied (bottom left). Binary image showing discrete objects that pass threshold value (bottom right).

It is often useful to identify objects within muscle sections that are not within fibers. The object counter was designed to identify capillary density using a PECAM staining to identify endothelial cells, however other cell types could be analyzed with the same techniques, such as macrophage infiltration [29]. The user is prompted for pixel size and the RGB channel of the objects to be identified (Figure 8A). Initial values for smoothing the objects, identical to that for nuclei and fiber type, with initial threshold automatically generated using Otsu’s method. The user also identifies the folder location of the Excel file output. After initial parameters are selected a figure is presented with a figure containing the original image, the object channel image, smoothed object channel image, and thresholded object image (Figure 8C). After inspection, the user is then prompted to change the threshold if desired. Once the user has achieved a suitable threshold the user selects whether to output an Excel file with the data. Each object has its area and centroid listed along with summary data including the total number of objects in the image.

### SMASH data verification

To verify the data generated by SMASH we compared the same images used in previously published data [30]. In this case legacy methods were done using Openlab software (Improvision, PerkinElmer) with simple thresholding of individual RGB channels for fiber size and fiber type. For central nucleation fibers were manually determined to be CNF or PNF and a marker placed using Openlab to enable counting.

### Statistical analysis

The C57 and *mdx* output data were compared using a Student’s t-test. For comparing CNFs and PNFs a paired Student’s t-test was used. All data analyses were performed using PRISM (Graphpad Software).

## Results

**Figure 9:**
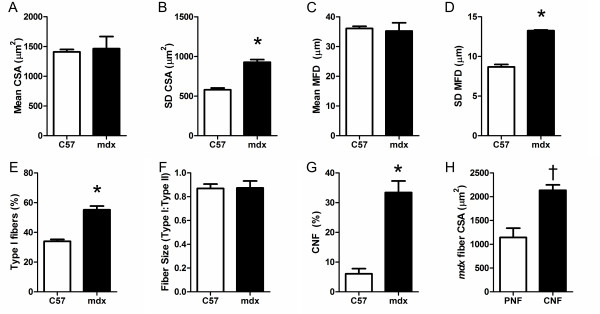
**Comparison of output data from 1-year-old C57 and** ***mdx*** **mouse soleus muscle. (A)** Mean of cross-sectional area of fibers in C57 and *mdx* muscle sections. **(B)** Standard deviation of CSA of fibers. **(C)** Mean of minimum Feret diameter (MFD) of fibers. **(D)** Standard deviation of MFD diameter. **(E)** Percentage of Type I fibers in C57 and *mdx* muscle sections. **(F)** The ratio of mean CSA of type I fibers to type II fibers. **(G)** The percentage of fibers with centrally nucleated fiber (CNF)s. **(H)** The mean CSA of *mdx* peripherally nucleated fibers (PNF) and CNFs. *P <0.05 for mdx compared to C57, † *P* <0.05 for CNFs compared to PNFs within the same muscle.

In order to evaluate the suitability of the software described, skeletal muscle morphologies from a selection of wild-type C57BL6 and *mdx* mouse soleus muscles were analyzed (n = 4 per group). The *mdx* mouse is a model of Duchenne muscular dystrophy (DMD) that is commonly studied. A multitude of previous reports demonstrate that mean individual fiber cross-sectional area in *mdx* muscle is not consistently altered. However, as the *mdx* muscle is undergoing continual cycles of degeneration and regeneration the variability in fiber CSA is much greater than wild-type muscle [2, 31]. The results produced here clearly reflect the previous literature on mean CSA and variability of CSA (Figure 9A and B). The mean fiber CSA was not different between the C57 and *mdx* muscles; however the standard deviation (SD) of CSA within a muscle was much greater in *mdx* than C57. While measurements of fiber CSA have commonly been used, the fiber CSA is highly dependent on the angle of the section relative to the axis of the fiber. Use of minimal Feret diameter provides a robust measure of fiber size largely independent of the angle of the section and is endorsed as a standard operating procedure [2]. Our results on Feret diameter align well with that of CSA showing no change in the mean Feret diameter, but a substantial increase in the standard deviation of Feret diameter within an *mdx* muscle (Figure 9C and D).

Fiber type determination is commonly performed in muscle histological analysis. The muscles studied here have been stained for slow myosin heavy chain (Type I). The soleus muscle has a large portion of type I fibers of approximately 40% in wild-type mice [32]. This proportion is close to the value obtained with the software (Figure 9E), however there was a large increase in the percentage of slow fibers in the *mdx* soleus muscle. This is expected based on the advanced age of these mice of 1 year and that type I fibers are more resistant to damage in *mdx* muscle [33]. Determination of the size of various fiber types is an automatic feature of the software. Assuming all unlabeled fibers are type II fibers the results show that C57 type I fibers are smaller than type II and the relationship is not different in *mdx* soleus muscles (Figure 9F).

Due to the continual cycles of degeneration and regeneration, dystrophic muscles have a high prevalence of CNFs, while uninjured wild-type muscles have very few CNFs. The percentage of CNFs in dystrophic muscle is a commonly measured histological marker of disease [34]. The automatic detection demonstrated the expected results with C57 soleus muscle showing very rare CNFs while >30% of *mdx* soleus muscle fibers were CNFs [33, 35] (Figure 9G). While CNF percentage is routinely performed, often using manual methods, reporting on the size of the CNFs is less common. The software makes it trivial to measure the size of the CNF population. The data collected show that within *mdx* muscle CNFs are larger than peripherally nucleated fibers (PNFs) (Figure 9H). This relationship has been previously observed in the EDL muscle of *mdx* mice [16, 36].

**Figure 10:**
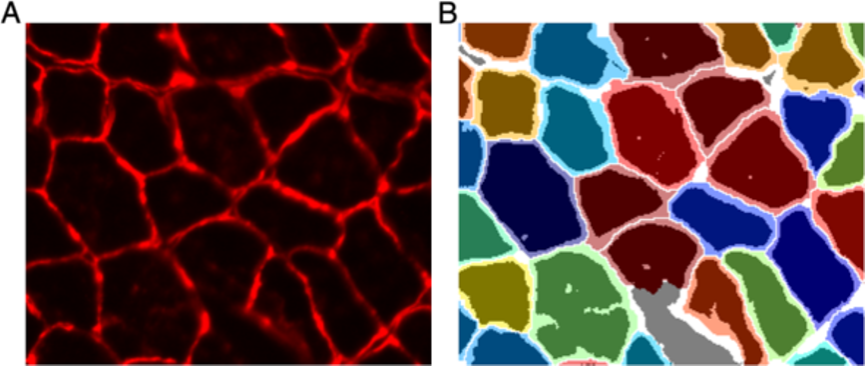
**Comparison of mask file from legacy method and SMASH. (A)** Original image of a laminin stained muscle section. **(B)** Fibers are colored randomly from SMASH output mask with dark regions corresponding to fiber area using a simple threshold. Dark gray areas are interstitial in SMASH and fibers using legacy methods while white areas are interstitial in both masks. Figure demonstrates the larger fiber area obtained with SMASH compared to the legacy method of using a simple threshold for fiber area.

The difference between data output from legacy methods and SMASH for fiber size, fiber size variability, fiber type percentage, and CNF percentage are presented in Table 2. There was little discrepancy between values obtained for fiber type percentage or CNF percentage. However, the calculated fiber areas were consistently higher using SMASH compared to legacy methods. This is due to the greater and more variable fiber border region using simple thresholding in legacy systems compared to SMASH (Figure 10). The time required to process each function in SMASH compared to legacy methods is reported in Table 3. Smash demonstrates greatly reduced time of analysis for the muscles tested.

**Table 2:** **Data comparison from legacy methods to SMASH**

Image	Legacy Method	SMASH	Difference
Fiber type - myosin IIa (%)			
A	59	58	-1
B	60	57	-3
C	58	61	3
Fiber size - fiber area (μm^2^)			
A	708	898	189
B	745	934	189
C	622	819	198
Fiber size - fiber area SD (μm^2^)			
A	303	415	112
B	265	346	81
C	219	339	121
Centrally nucleated fibers (%)			
A	71	66	-5
B	58	55	-3
C	61	60	-1

**Table 3:** **Run time (mm:ss) comparisons from legacy methods to SMASH**

Image	Legacy method - functions				SMASH - functions						Diff
	*FP* ^a^	*FT* ^a^	*CNF*	*TOT*	*IS*	*FF*	*FP*	*FT*	*CNF*	*TOT*	*TOT*
A	30:	10:	16:30	56:30	1:50	1:49	0:06	0:17	0:12	4:14	52:16
B	30:	10:	13:50	53:50	2:46	3:42	0:05	0:26	0:15	7:14	46:36
C	30:	10:	13:30	53:30	1:45	2:39	0:07	0:14	0:10	4:55	48:35

^a^Times are approximate.

CNF functions were performed on different sections than other functions.

CNF: Centrally nucleated fibers function; FF: Fiber filter function; FP: Fiber properties function, including fiber size; FT: Fiber typing function; IS: Initial segmentation function; TOT: Total time for analysis.

## Discussion

The manual analysis of skeletal muscle immunofluorescence is often a laborious task. The Semiautomatic Image Processing of Skeletal Muscle Histology Software described and tested provides researchers with a valuable tool for measuring multiple facets of muscle histology. While image analysis software is available to conduct many of these features, it carries a high monetary cost and is not specifically designed for skeletal muscle analysis. In contrast, this program is available on the widely used MATLAB software and is designed to investigate specific features of muscle histology, and is open to custom modifications for advanced users.

All of the functions in the program rely and are based on the segmentation of skeletal muscle fibers in an image. This is commonly done with manual tracing, which is very time-consuming, or basic thresholding of fiber outlines, which requires extensive manual correction on all but the most pristine sections. Using a combination of the image smoothing fiber and the watershed transform fiber boundaries are automatically produced with much improved reliability over standard thresholding. However, the software maintains the manual ability to correct image segmentation. Furthermore, if the built-in manual editing features are not suitable, the user may edit the created mask file with software of his choice, such as Adobe Photoshop. After the mask has been finalized, running the analysis functions is expedient. The user is required to enter parameters for analysis and may adjust them based on the resulting image guides. To analyze the entire cross-section of 1-year-old soleus muscles for fiber size, fiber type, and CNFs from the same image took approximately 15 min, substantially less than manual methods. Increased time efficiency allows analysis of the whole muscle cross-section, avoiding the sampling issues of only using select fields of view. Using the whole muscle cross-section is required to meet TREAT-NMD standards of practice [37]. Analyzing more fiber and eliminating regional differences in cross-section improves the consistency of the results. Highly significant results were obtained with low variability in our investigation with only four muscles per group.

The output of fiber size data in Excel format permits the use of many graphing tools to create plots of fiber size commonly used. Ensuring the ability to measure Feret diameter ensures compliance with the latest standard operating procedures for fiber size analysis [2]. Automation of fiber type data with defined boundaries has advantages over using thresholding to determine fiber type and size. Instead of determining the fiber size by the area above a threshold value of staining, the area is determined entirely by the fiber outline so the positive fiber size will not be affected by blotchiness in fiber type staining. The fiber type function may also be used for additional signals internal to fibers such as Evan’s Blue Dye permeability or IgG infiltration of necrotic fibers [2, 38]. Automatic determination of CNFs provides a useful measure of skeletal muscle histology. Although it is not frequently studied, combining fiber size with CNF determination may provide a more informative marker of muscle health [16]. The computation of this additional parameter is trivial using this software. Analysis of structures outside of fibers themselves in skeletal muscle is also important. The object counter function was designed to automate analysis of capillary density, as is done in muscle histological analysis for capillaries per fiber and capillaries per area [19]. However, it may also be used to analyze a multitude of other structures within a muscle, such macrophage infiltration or matrix proteins.

The data generated by SMASH are validated against legacy methods showing largely consistent results between methods for fiber type and CNF percentages (Table 2). However, the discrepancy in fiber area illustrates the importance of using a single analytical method for a given study and highlights the variability between different approaches. Using a simple threshold to separate fibers creates highly variable borders between adjacent fibers that are dependent on parameters such as exposure time and focus during image acquisition. Filtering the signal and using the watershed function as is done in SMASH provides signal that is more robust to these parameters. SMASH provides a mask more consistent with manual tracing of fibers than applying a threshold. While SMASH reduces the border region between adjacent fibers, it is also clearly capable of delineating interstitial space between adjacent fibers when there is an appreciable separation as evidenced by the white and grey areas in Figure 10. Thus, we attest that SMASH generally provides a more robust and accurate fiber size as well as requiring less manual editing than using simple thresholds. It is also noteworthy that fiber area is generally more variable than Feret diameter as for perfectly round areas it is squaring the difference and the more elongated the fiber the greater the proportion of border region that may influence the results.

In addition the gains in robust analysis Table 3 demonstrates that SMASH greatly reduces the time required to analyze images. Manual editing of the fiber mask is still required in the majority of muscle sections for both initial segmentation and fiber filtering and take the majority of the processing time. However, with SMASH this manual editing is reduced to just a few minutes in the case of the soleus muscles tested. The time gains are especially significant when doing multiple analyses on the same image, as manually editing the mask is the major time consumer and additional functions are able to be processed in just seconds.

While this software provides many advantages there are notable limitations. The analysis is currently limited to immunofluorescence and is not compatible with common stains such as hematoxylin and eosin. The software does often require manual segmentation and filtration of fibers based on the current algorithms. These manual adjustments allow the user more control over the analysis, but also increase the time required for analysis and introduce a degree of subjectivity. Manual adjustments are more frequently required in analysis of diseased muscle as well. While these algorithms are advanced compared to many of the techniques currently in use, extending fiber segmentation algorithms may provide more reliable boundaries. The proposed method of measuring fiber type is limited to investigation of a single fiber type per image, or per color in an RGB image. Thus to measure each fiber type individually requires multiple image segmentation masks from serial sections. Alternatively, using distinguishable fluorophors for each fiber type [39] would permit the analysis of two fiber types using the same mask using this software. Using serial sections for fiber typing is standard procedure in many labs and it is not enhanced by this software. The analysis of myonuclei is limited to CNFs in this software, as opposed to providing a measure of myonuclear density with peripheral nuclei [9, 20]. The method of measuring CNFs is recommended by TREAT-NMD, however it causes an issue with very small fibers as the entire CSA may be in the defined border region, making it impossible to be labeled as a CNF. The measurement of non-fiber objects designed for capillary density is currently preliminary. There is no filtering of objects or a method to select an object of interest. However, the output of object size does allow filtering based on the objects CSA within Excel. This program currently is designed for use with muscle cross-sections and not designed to analyze images from longitudinal muscle sections. As an open source program, users may address any of these limitations as they see fit within the framework of the software platform and MATLAB.

## Conclusions

The software package based in MATLAB provides image processing tools to analyze immunofluorescent muscle cross-sections. The semi-automatic fiber segmentation functions provide advanced algorithms for fiber segmentation as well as provide an interface for users to manually correct any errors. The histological analysis includes functions for fiber CSA, fiber Feret diameter, fiber typing, CNFs, and capillary density. These functions produced expected results comparing wild-type and dystrophic mouse muscle. These functions may be purposed for other analyses. This open source platform provides users a framework to create their own functions or modification of previously incorporated functions. Automated functions improve the speed and consistency of skeletal muscle histological analysis. Although it requires a MATLAB license, this is the only freely available software designed for the analysis of skeletal muscle histology.

## Availability and requirements

**Project name:** SMASH - Semiautomatic Image Processing of Skeletal Muscle Histology: a MATLAB Application.

**Project homepage:**http://dx.doi.org/10.6084/m9.figshare.1247634

**Operating System:** Platform Independent.

**Programming Language:** MATLAB.

**Other requirements:** SMASH Stand Alone (SMASH_Installer.exe) requires MATLAB Compiler Runtime (MCR) version R2014a (8.3) which is freely available from Mathworks (http://www.mathworks.com/products/compiler/mcr/).

SMASH App (SMASH_App.mlappinstall) requires MATLAB version R2014a (8.3) or later with the Image Processing Toolbox.

**License:** CC-BY.

**Any restrictions to use by non-academics:** None.

## Abbreviations

CNF: Centrally nucleated fiber
CSA: Cross-sectional area
DAPI: 4′,6-diamidino-2-phenylindole
EDL: Extensor digitorum longus
PECAM: Platelet endothelial cell adhesion molecule
PNF: Peripherally nucleated fiber
RGB: Red, Green, and Blue image format.
